# A blockade of PI3Kγ signaling effectively mitigates angiotensin II-induced renal injury and fibrosis in a mouse model

**DOI:** 10.1038/s41598-018-29417-3

**Published:** 2018-07-20

**Authors:** Xinyi Yu, Yunfeng Xia, Liyi Zeng, Xi Zhang, Liqun Chen, Shujuan Yan, Ruyi Zhang, Chen Zhao, Zongyue Zeng, Yi Shu, Shifeng Huang, Jiayan Lei, Chengfu Yuan, Linghuan Zhang, Yixiao Feng, Wei Liu, Bo Huang, Bo Zhang, Wenping Luo, Xi Wang, Hongmei Zhang, Rex C. Haydon, Hue H. Luu, Tong-Chuan He, Hua Gan

**Affiliations:** 1 Departments of Nephrology, Orthopaedic Surgery, Cardiology, General Surgery, and Clinical Laboratory Medicine, the First Affiliated Hospital of Chongqing Medical University, Chongqing, 400016 China; 2 Molecular Oncology Laboratory, Department of Orthopaedic Surgery and Rehabilitation Medicine, The University of Chicago Medical Center, Chicago, IL 60637 USA; 3 Department of Infection Control, Zhuzhou Central Hospital, and the Affiliated Zhuzhou Hospital of Xiangya Medical College of Central South University, Zhuzhou, China; 4 Ministry of Education Key Laboratory of Diagnostic Medicine and School of Laboratory Medicine, and the Affiliated Hospitals of Chongqing Medical University, Chongqing, 400016 China; 5 Department of Biochemistry and Molecular Biology, China Three Gorges University School of Medicine, Yichang, 443002 China; 6 Department of Clinical Laboratory Medicine, the Second Affiliated Hospital of Nanchang University, Nanchang, 330031 China; 7 Key Laboratory of Orthopaedic Surgery of Gansu Province and the Department of Orthopaedic Surgery, the Second Hospital of Lanzhou University, Lanzhou, 730030 China; 8 Chongqing Key Laboratory for Oral Diseases and Biomedical Sciences, and the Affiliated Hospital of Stomatology of Chongqing Medical University, Chongqing, 401147 China

## Abstract

Chronic kidney disease (CKD) poses a formidable challenge for public healthcare worldwide as vast majority of patients with CKD are also at risk of accelerated cardiovascular disease and death. Renal fibrosis is the common manifestation of CKD that usually leads to end-stage renal disease although the molecular events leading to chronic renal fibrosis and eventually chronic renal failure remain to be fully understood. Nonetheless, emerging evidence suggests that an aberrant activation of PI3Kγ signaling may play an important role in regulating profibrotic phenotypes. Here, we investigate whether a blockade of PI3Kγ signaling exerts any beneficial effect on alleviating kidney injury and renal fibrosis. Using a mouse model of angiotensin II (Ang II)-induced renal damage, we demonstrate that PI3Kγ inhibitor AS605240 effectively mitigates Ang II-induced increases in serum creatinine and blood urea nitrogen, renal interstitial collagen deposition, the accumulation of ECM proteins and the expression of α-Sma and fibrosis-related genes *in vivo*. Mechanistically, we reveal that AS605240 effectively inhibits Ang II-induced cell proliferation and phosphorylation of Akt in fibroblast cells. Furthermore, we demonstrate that Ang II-upregulated expression of IL-6, Tnf-α, IL-1β and Tgf-β1 is significantly attenuated in the mice treated with AS605240. Taken together, our results demonstrate that PI3Kγ may function as a critical mediator of Ang II-induced renal injury and fibrosis. It is thus conceivable that targeted inhibition of PI3Kγ signaling may constitute a novel therapeutic approach to the clinical management of renal fibrosis, renal hypertension and/or CKD.

## Introduction

Chronic kidney disease (CKD) is a group for heterogeneous disorders affecting kidney structure and function presenting as a growing public health problem worldwide^1–3^. Risk factors for CKD development and progression include low nephron number, nephron loss and kidney injury caused by toxic exposures or diseases such as obesity and diabetes^4,5^. Nonetheless, it has also recognized that genetic polymorphisms and epigenetic variations determine the individual susceptibility of patients to develop chronic progressive kidney disease^6^. Clinical manifestations of kidney dysfunction include hypertension, edema, changes in output or quality of urine and growth delay, and increased serum levels of creatinine, blood urea nitrogen, cystatin C and uremia toxins as well as lipid dysregulation^7–9^. Regardless of the initiating insults or contributing risk factors, the common pathological features in patients with CKD are inflammation, tubular atrophy, and renal interstitial fibrosis^10–14^.

Fibrogenesis is a pathological scarring process involving the accumulation of activated fibroblasts, excessive deposition of extracellular matrix, failed regeneration of tubular epithelium, microvascular rarefaction and inflammation^6,15^. Renal fibrosis is the final common manifestation of chronic kidney disease that leads to end-stage renal disease^10,16,17^. In fact, the extent of tubulointerstitial fibrosis is the best predictor for kidney survival, irrespective of the underlying disease^6^. Thus, tubulointerstitial fibrosis is considered the common pathway of chronic progressive CKD^6^. Tubulointerstitial fibrosis is characterized by fibroblast activation and excessive production and deposition of extracellular matrix (ECM), resulting in the destruction of renal parenchyma and causes progressive loss of kidney function^16,18–20^. However, the pathogenesis and the initial molecular events leading to tubulointerstitial fibrosis and eventually chronic renal failure remain to be fully understood. Thus, there is an unmet clinical challenge to fully understand the cellular and molecular mechanisms underlying the pathogenesis of CKD^21^. Nonetheless, it has been well-recognized that activation of the renin–angiotensin system (RAS) plays a central role in initiation and progression of CKD through regulation of inflammation and fibrosis^22,23^. Recent studies have shown that angiotensin II (Ang II) contributes to tubulointerstitial fibrosis by TGF-β1/Smad including Smad-dependent and Smad-independent signaling pathways^24^. However, the detailed mechanism underlying Ang II-induced kidney injuries are not fully understood.

Phosphoinositide 3-kinases (PI3Ks) contributed to Smad-independent signaling pathway are a subfamily of lipid kinases that play an important role in intracellular signaling and are involved in many homeostatic mechanisms. PI3Ks are divided into three classes^25^. Originally thought to be relevant for several leukocyte functions, PI3Kγ is the unique member of class IB, which is activated by G protein-coupled receptors (GPCRs) via binding to βγ subunits^25^, and expressed in leukocytes and other cell types, including endothelial cells and fibroblasts^26^. It was shown that mice lacking PI3Kγ were protected from hypertension induced by Ang II^27^, and resistant to bleomycin-induced pulmonary injury, angiogenesis and fibrosis^28^. Thus, these studies strongly suggest that an aberrant activation of PI3Kγ signaling may play an essential role in regulating profibrotic phenotypes.

In this study, we investigate whether a blockade of PI3Kγ signaling exerts any beneficial effect on alleviating kidney injury and tubulointerstitial fibrosis by using an Ang II-induced renal damage mouse model. Our results demonstrate that PI3Kγ may function as a critical mediator of Ang II-induced renal injury and fibrosis. It is thus conceivable that targeted inhibition of PI3Kγ signaling may be explored as a novel therapeutic approach to the clinical management of renal fibrosis, renal hypertension and/or CKD.

## Materials and Methods

### Cell culture and chemicals

Mouse fibroblast line NIH-3T3 cells were obtained from ATCC (Manassas, VA) and maintained in complete Dulbecco’s Modified Eagle Medium(DMEM) containing 10% fetal bovine serum (Invitrogen, Carlsbad, CA), 100 units of penicillin and 100 µg of streptomycin at 37 °C in 5% CO_2_. Ang II was purchased from Sigma-Aldrich (St. Louis, MO). PI3Kγ inhibitor AS605240 was purchased from Selleckchem (Huston, TX). Akt and p-Akt antibodies were obtained from Santa Cruz Biotechnology (Dallas, TX); while Col1a, fibronectin, α-Sma and Gapdh antibodies were purchased from Abcam (Cambridge, MA). Unless otherwise indicated, all chemicals were purchased from Sigma-Aldrich (St. Louis, MO) or Thermo-Fisher Scientific (Pittsburgh, PA).

### Crystal violet cell viability assay

Crystal violet assay was conducted as described^29,30^. Briefly, subconfluent NIH-3T3 cells were treated with AS605240 and/or Ang II or DMSO. At 48 h after treatment, cells were carefully washed with PBS and stained with 0.5% crystal violet/formalin solution at room temperature for 20–30 min. The stained cells were washed with tape water and air dried for taking macrographic images. For quantitative measurement, the stained cells were dissolved in 10% acetic acid (1 ml per well for 12-well plate) at room temperature for 20 min with shaking. 500 µl were taken and added to 2 ml ddH_2_O. Absorbance at 570–590 nm was measured^31,32^.

### Mouse model of Ang II-induced hypertension and renal injury

Animal care and use of the reported study were approved by the Ethics Committee of Chongqing Medical University and in compliance with the guidelines for the Care and Use of Laboratory Animals by the National Research Council. Male C57BL/6 mice were purchased from and housed in the Experimental Animal Center of Chongqing Medical University. The animals were maintained at optimal temperature with a 12:12 h light-dark cycle and free access to food and water. Experimentally, 15 male mice (8–10 week-old) were subjected to uninephrectomy^33^, and randomly divided into three groups (n = 5 per group): the control group, which did not receive any further treatment; the Ang II only group, which received a continuous Ang II infusion (1.5 µg/kg/min, Sigma) via subcutaneous osmotic mini-pumps (Alzet) and oral administration of normal saline; and the Ang II + AS605240 group, which received continuous infusion of Ang II (1.5 µg/kg/min, Sigma) via subcutaneous osmotic mini-pumps (Alzet) and orally administered (via gavage) with AS605240 at 50 mg/(kg·d). All animals were euthanized at 28 days after treatment. At the endpoint, the blood samples were collected. The animals were perfused with PBS and the kidney tissues were retrieved for total RNA and protein isolations, as well as for histological analysis (see below).

### Renal function analysis

Approximately 0.2 ml of blood was collected from each mouse. Briefly, mice were anesthetized, and blood was collected by using the retro-orbital bleeding approach. Sera were prepared from the collected blood samples and stored at −80 °C prior to assays. Blood urea nitrogen and serum creatinine were measured using the clinical sample assay kits obtained from Nanjing Jiancheng Bioengineer Institute (Nanjing, China) according to the manufacturers’ instructions.

### Histopathologic analysis

A portion of the retrieved mouse kidney tissue was fixed in 4% buffered formalin, embedded in paraffin, and sectioned^34^. After deparaffinization and rehydration, kidney tissue sections were subjected to Masson Trichrome (Sigma-Aldrich) and Picrosirius Red (Abcam, Cambridge, MA) staining by following the manufacturers’ instructions. The staining results were recorded under a bright field microscope. For the quantitative analysis, at least 10 random high-power fields (i.e., 200x) were selected and evaluated by using Image Pro Plus 6.0 software. The positive area was calculated as a % of the total area.

### Immunohistochemical (IHC) staining

The IHC staining was carried out as described^35–37^. Briefly, mouse kidney tissues were paraffin-embedded, sectioned, and deparaffinized. After deparaffinization, rehydration and antigen retrieval, sections were blocked and incubated with rabbit anti-Col1a antibody (Abcam), rabbit anti-fibronectin antibody (Abcam), or rabbit anti-α-Sma antibody (Abcam), followed by incubation with a biotin-labeled goat anti-rabbit secondary antibody and streptavidin-conjugated horseradish peroxidase (HRP). The protein of interest was visualized by 3,3′-diaminobenzidine (DAB) staining^38–41^. Rabbit IgG and minus primary antibody stains were used negative controls. Quantitative evaluation of staining results was performed using Image Pro Plus 6.0 software by examining >10 random high-power fields (i.e., 200x magnification) of each sample. The average positive staining area was calculated as the percentage of the total area.

### Quantitative real-time PCR (qPCR) analysis

Total RNA was extracted from kidney tissues with Nucleozol reagent (Takara, Japan and USA) and reverse transcribed with Prime Script RT reagent kit (Takara, Japan) or using hexamer and M-MuLV reverse transcriptase (New England Biolabs, Ipswich, MA). The resulting cDNA products were diluted and used as PCR templates. TqPCR was carried out by using SYBR Green-based TqPCR analysis on a CFX-Connect unit (Bio-Rad Laboratories, Hercules, CA). PCR primers were designed by using Primer3 program and were listed in Supplementary Table 1. Quantitative real-time PCR analysis was carried out by using our recently optimized TqPCR protocol^42–44^. Briefly, the PCR reactions were carried out by using a touchdown protocol: 95 °C × 3 min for one cycle; 95 °C × 20 sec, 66 °C × 10 sec for 4 cycles, with 3 °C decrease per cycle; followed by 95 °C × 10 sec, 55 °C × 15 sec, 70 °C × 1 sec for 40 cycles, followed by plate read. All reactions were done in triplicate. The TqPCR amplification was confirmed by performing the melting curve test and observing a single peak for each gene. *Gapdh* was used as a reference gene. The comparative Ct method (ΔΔCt) was used to quantify gene expression, and the relative quantification was calculated as 2−ΔΔCt.

### Western blotting analysis

Western blotting assay was carried out as described^45,46^. Briefly, tissue or cell lysates were prepared in Laemmli Sample Buffer. Equal amounts of total proteins were subjected SDS-PAGE and transferred to PVDF membranes, which were blocked and incubated overnight with the primary antibodies against Akt (Santa Cruz Biotechnology), p-Akt (Santa Cruz Biotechnology), Col1a (Abcam), fibronectin (Abcam), α-Sma (Abcam), or Gapdh (Abcam). After being washed, the membranes were incubated with respective secondary antibodies conjugated with horseradish peroxidase (HRP). Immune-reactive signals were visualized by the Enhanced Chemiluminescence (ECL) kit (Millipore, America) on Syngene PXi6 Access imaging system (Frederick, MD). The band intensities were quantified using Image Pro Plus 6.0.

### Statistical analysis

All data were expressed as mean ± SEM. Multiple group comparisons were performed by one-way ANOVA followed by the Bonferroni procedure for comparison of means. P < 0.05 was considered statistically significant.

## Results

### Inhibition of PI3Kγ signaling improves renal functions in a mouse model of Ang II-induced renal injury

**Figure 1:**
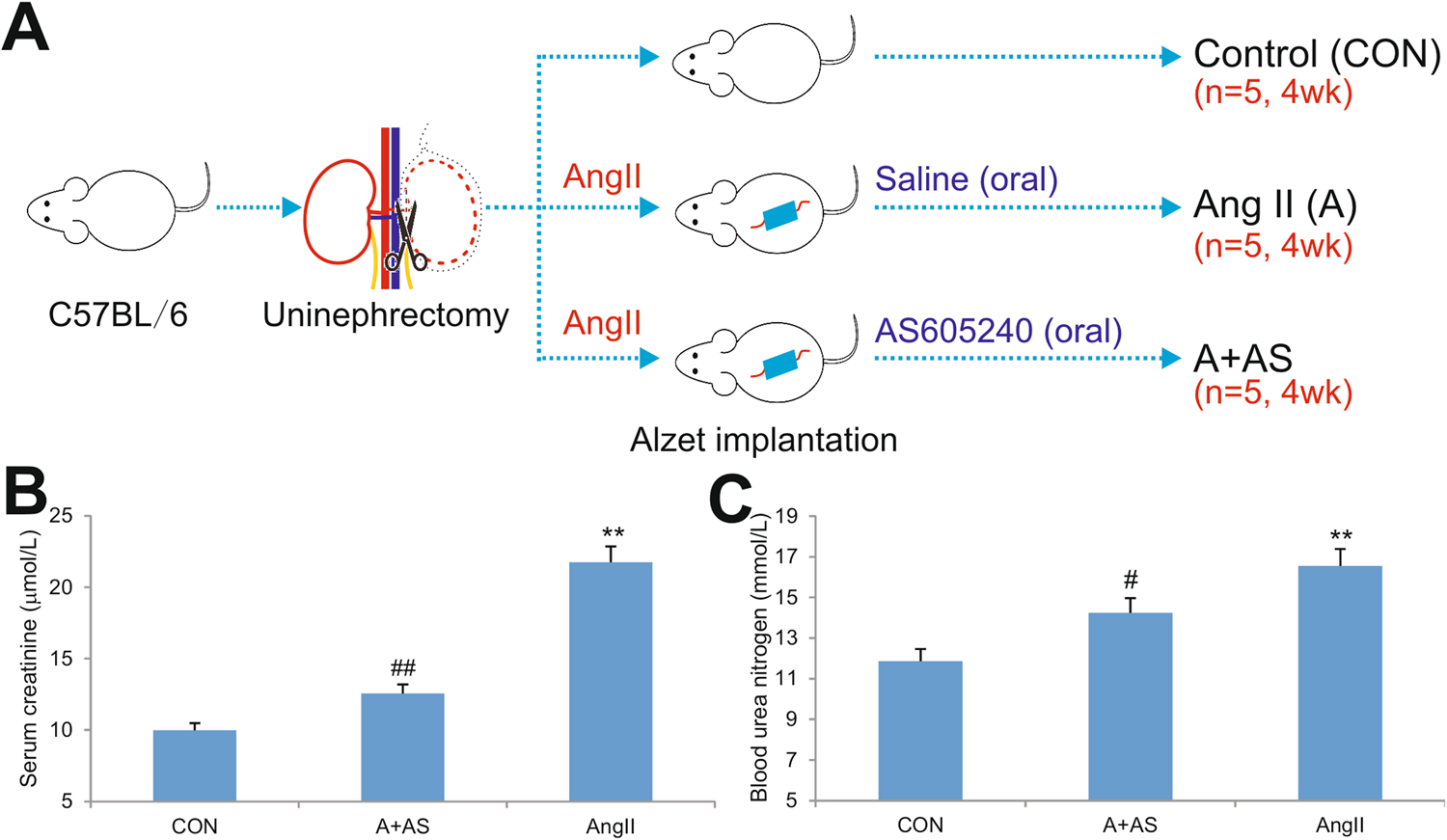
Inhibition of PI3Kγ signaling improves renal functions in a mouse model of Ang II-induced renal injury. (**A**) Schematic representation of the establishment of renal injury mouse model by continuous infusion of Ang II via subcutaneous osmotic mini-pumps with or without oral administration of AS605240 (n = 5 per group). (**B**) Ang II-induced elevation of serum creatinine is significantly reversed by PI3Kγ inhibition. (**C**) Ang II-induced elevation of blood urea nitrogen is effectively reversed by PI3Kγ inhibition. ^**^p < 0.01, compared with that of the control group; ^#^p < 0.05, ^##^p < 0.01, compared with that of the Ang II treatment group.

To test whether a blockade of PI3Kγ activity would alleviate kidney damage caused renal hypertension, we utilized the well-established animal model of angiotensin II-induced hypertension, in which the mice were first performed with uninephrectomy and randomly divided into the control group, which did not receive any further treatment, the Ang II only group, which received a continuous Ang II infusion (1.5 µg/kg/min) via subcutaneous osmotic mini-pumps (Alzet) and oral administration of normal saline, and the Ang II + AS605240 group, which received continuous infusion of Ang II (1.5 µg/kg/min) via subcutaneous Alzet osmotic mini-pumps along with oral administration of AS605240 (50 mg/kg/day). All animals were euthanized at 28 days after treatment (Fig. 1A). The animal model of Ang II-induced renal injury was successfully established as the serum level of creatinine and blood urea nitrogen were significantly elevated in the Ang II treatment group, compared with that of the control group (p < 0.01) (Fig. 1B,C). However, Ang II-induced increases in serum creatinine and blood urea nitrogen were significantly mitigated by PI3Kγ inhibitor AS605240 (p < 0.01 and p < 0.05 respectively) (Fig. 1B,C), suggesting that PI3Kγ blockade may be beneficial to preservation of kidney functions during renal hypertension.

### Inhibition of PI3Kγ activity alleviates Ang II-induced renal injury and fibrosis

**Figure 2:**
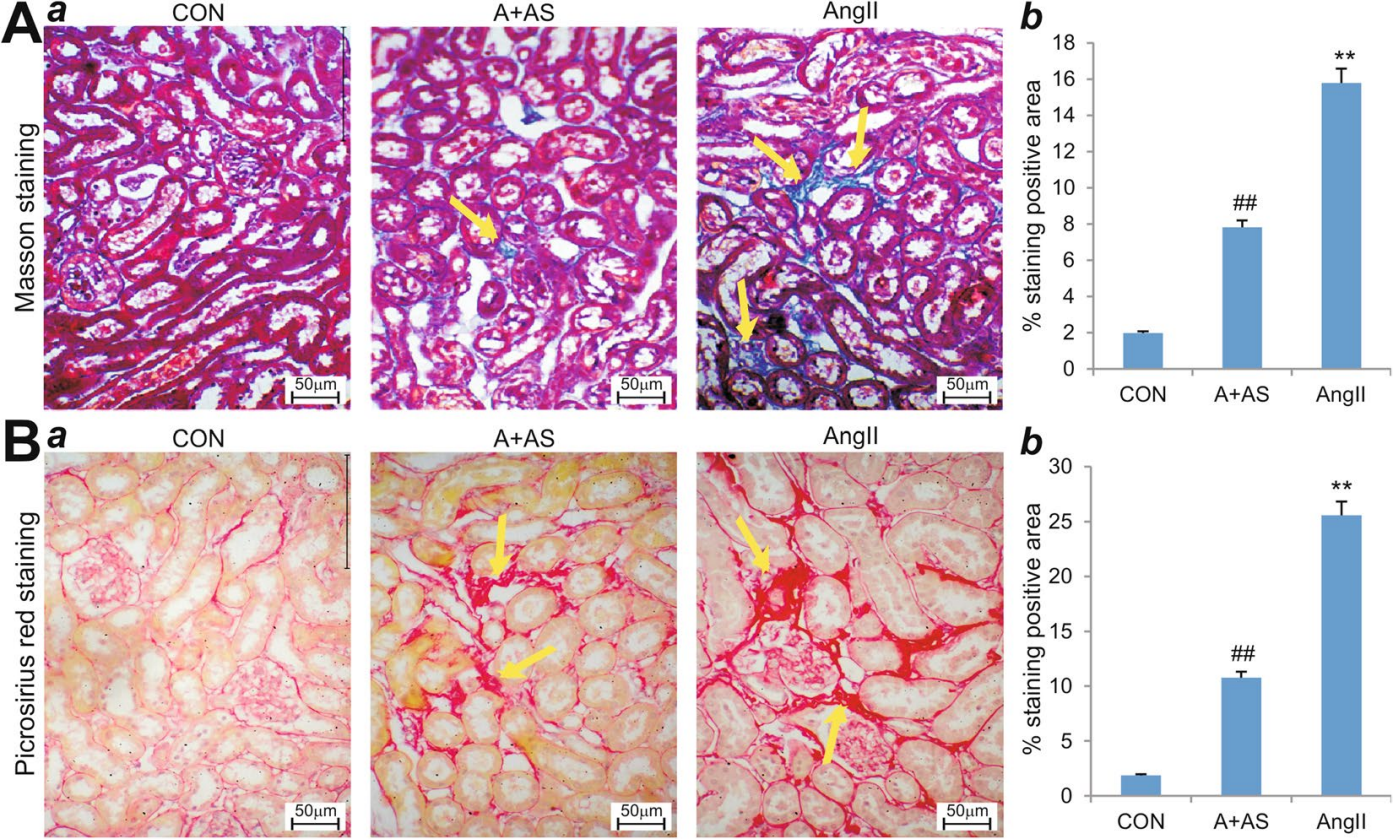
Inhibition of PI3Kγ activity alleviates Ang II-induced renal injury and fibrosis. (**A**) Masson trichrome staining of the kidney tissues retrieved from Ang II-treated mice with or without AS605240 (n = 5 per group) (a). Representative images are shown. Masson-positive interstitial collagen regions were quantitatively analyzed by using Image Pro Plus 6.0 (b). (**B**) Picrosirius Red staining of the retrieved kidney tissues from Ang II-treated mice with or without AS605240 (n = 5 per group) (a). Representative images are shown. Picrosirius Red-positive interstitial collagen regions were quantitatively analyzed by using Image Pro Plus 6.0 (b). Positive stains are indicated by yellow arrows. ^**^p < 0.01, compared with that of the control group; ^##^p < 0.01, compared with that of the Ang II treatment group.

We further examined the histopathologic features of the kidney tissues retrieved from the animal model experiments. Masson and Pircosirius Red staining revealed that Ang II treatment led to a significant increase in interstitial collagen deposition in the kidney, compared with the control group (p < 0.01) (Fig. 2A,B). However, such renal fibrotic response was significantly subdued in the AS605240-treated mice with chronic Ang II infusion (p < 0.01) (Fig. 2A,B).

**Figure 3:**
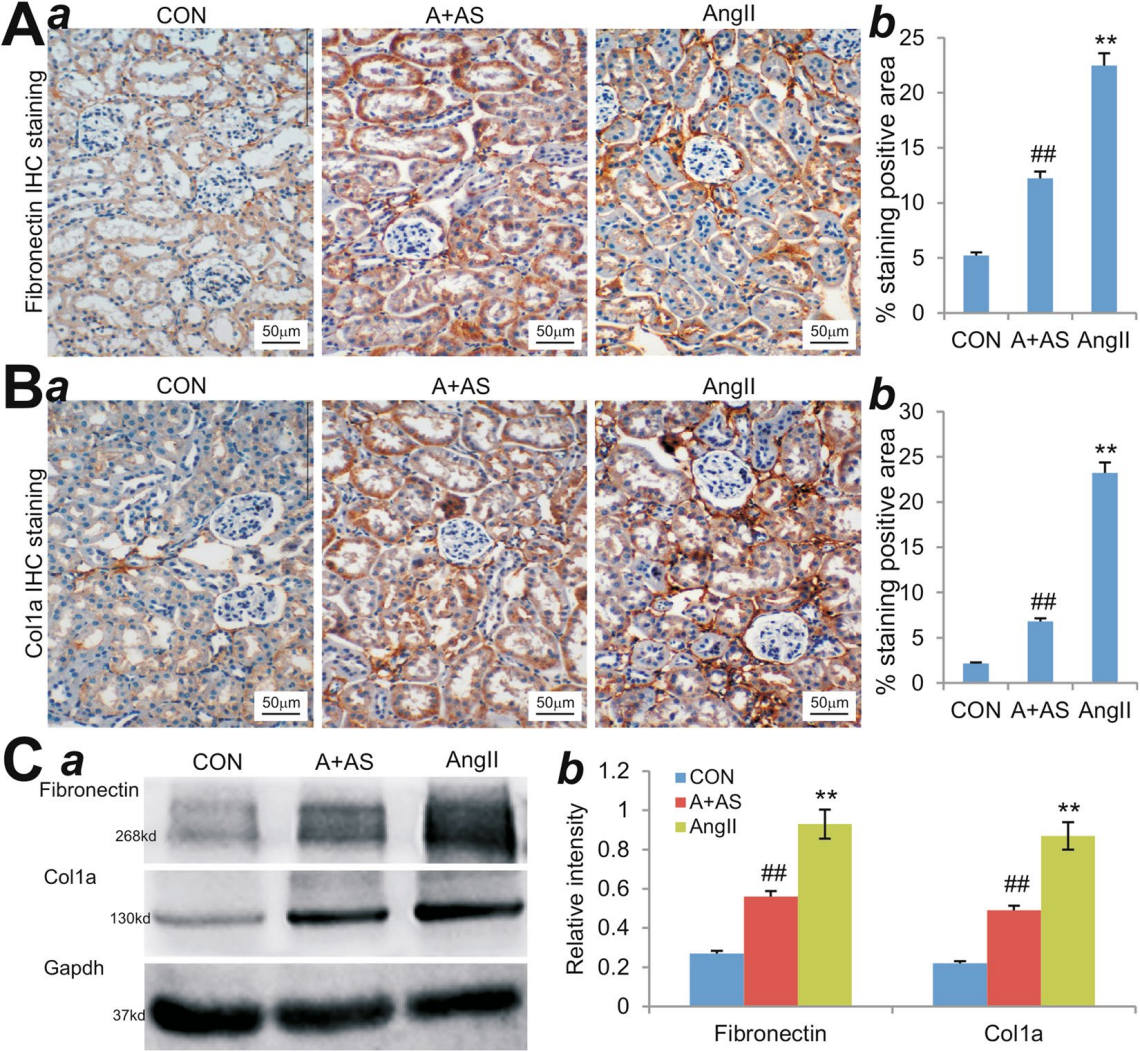
PI3Kγ inhibitor AS605240 suppresses Ang II-induced expression of fibronectin and type I collagen *in vivo*. (**A**,**B**) Immunohistochecmial (IHC) staining. The mouse kidney tissues retrieved from Ang II-treated mice with or without AS605240 (n = 5 per group) were subjected to immunohistochemical staining with fibronectin (**A**) or Col1a antibody (**B**). Representative IHC images are shown (a). Average positively-stained areas were quantitatively assessed with Image Pro Plus 6.0 (b). Isotopic IgG or no primary antibodies were used as negative controls (data not shown). (**C**) Western blotting analysis. The tissue lysates from the retrieved muse kidney tissues were subjected to SDS-PAGE and Western blotting with fibronectin, Col1a, or Gapdh antibody (a). The intensities of the blotting results were quantitatively determined by using Image Pro Plus 6.0 (b). ^**^p < 0.01, compared with that of the control group; ^##^p < 0.01, compared with that of the Ang II treatment group.

We further examined the effect of AS605240 on the expression of two major components of extracellular matrix and the hallmarks of fibrosis, fibronectin and type I collagen. Our immunohistochemical results indicated that Ang II treatment led to a significant increase in the expression of interstitial fibronectin (Fig. 3A) and type I collagen (Fig. 3B) in the mouse kidney, compared with that of the control group (p < 0.01). However, AS605240 treatment effectively mitigated the Ang II-upregulated expression of fibronectin (Fig. 3A) and type I collagen (Figure 3B) (p < 0.01). Moreover, Western blotting analysis confirmed that Ang II-induced expression of fibronectin (Fig. 3A) and type I collagen was significantly down-regulated by AS605240 (Fig. 3C). Collectively, the above results further support a potential protective effect of PI3Kγ inhibitor AS605240 on renal functions during renal hypertension.

### Inhibition of PI3Kγ signaling diminishes Ang II-induced expression of myofibroblast and fibrosis related genes *in vivo* and *in vitro*

**Figure 4:**
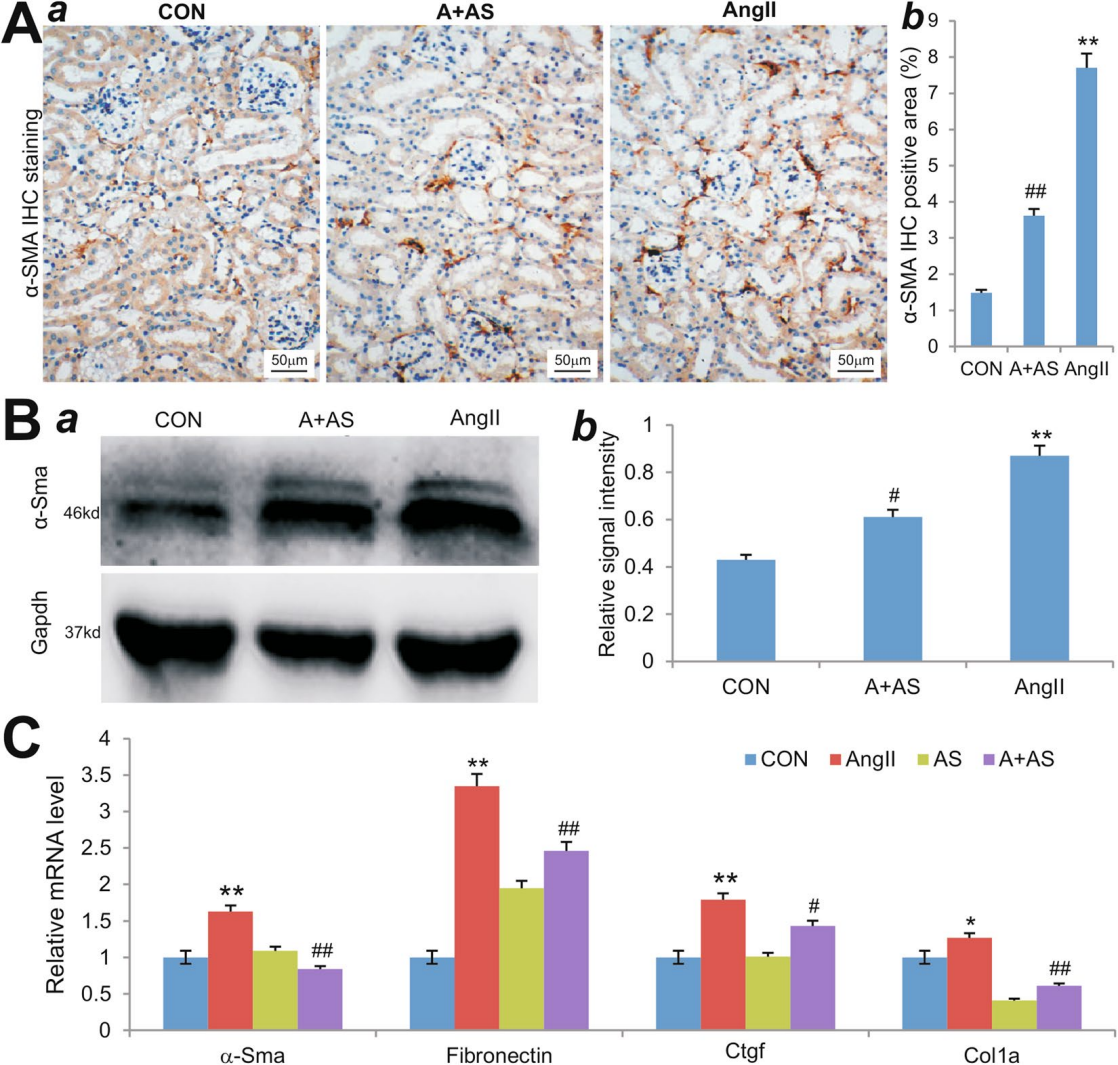
Inhibition of PI3Kγ signaling diminishes Ang II-induced expression of myofibroblast and fibrosis related genes *in vivo* and *in vitro*. (**A**) IHC staining of α-Sma expression. The kidney tissues retrieved from Ang II-treated mice with or without AS605240 (n = 5 per group) were subjected to immunohistochemical staining with α-Sma antibody. Representative IHC images are shown (a). Average positively-stained areas were quantitatively assessed with Image Pro Plus 6.0 (b). Isotopic IgG or no primary antibodies were used as negative controls (data not shown). (**B**) Western blotting analysis. The tissue lysates from the retrieved mouse kidney tissues were subjected to SDS-PAGE and Western blotting with α-Sma antibody or Gapdh antibody (a). The intensities of the blotting results were quantitatively determined by using Image Pro Plus 6.0 (b). (**C**) Quantitative PCR analysis of gene expression. Subconfluent NIH3T3 cells were treated with Ang II (A,1 µM), AS605240 (AS, 10 µM), Ang II (1 µM) and AS (10 µM) (A + AS), or DMSO control (CON) for 48 h. Total RNA was isolated from the treated cells and subjected to TqPCR analysis for the expression of α-Sma, fibronection, Ctgf and Col1a. All reactions were done triplicate. All samples were normalized with Gapdh expression levels. ^*^p < 0.05 and ^**^p < 0.01, compared with that of the control group; ^#^p < 0.05 and ^##^p < 0.01, compared with that of the Ang II treatment group.

We also examined whether PI3Kγ blockade would affect myofibroblast formation. When the retrieved mouse kidney sections were stained for the expression of the myofibroblast marker α-Sma, we found that AS605240-treated mice exhibited a significant reduction in the number of α-Sma-positive myofibroblasts in the kidneys when compared with that of the Ang II-treated mice (p < 0.01) (Fig. 4A). Consistent with the IHC staining results, Western blotting analysis revealed that blockade of PI3Kγ activity by AS605240 reduced the α-Sma expression level of in the kidneys retrieved from Ang II treatment (p < 0.01) (Fig. 4B).

Accordingly, we demonstrated that Ang II treatment significantly up-regulated the expression of myofibroblast marker α-Sma and fibrosis-related genes fibronectin, Ctgf and to a lesser extent Col1a in NIH3T3 cells *in vitro* (Fig. 4C). However, the Ang II-induced expression of the above genes was effectively suppressed by AS605240 (Fig. 4C). Taken together, these results further demonstrate that PI3Kγ blockade may provide protective effects in renal hypertension by suppressing the expression of fibrosis-related genes in kidney.

### Inhibition of PI3Kγ signaling suppresses Ang II-promoted fibroblast proliferation and blocks Ang II-activated Akt signaling in fibroblast cells

**Figure 5:**
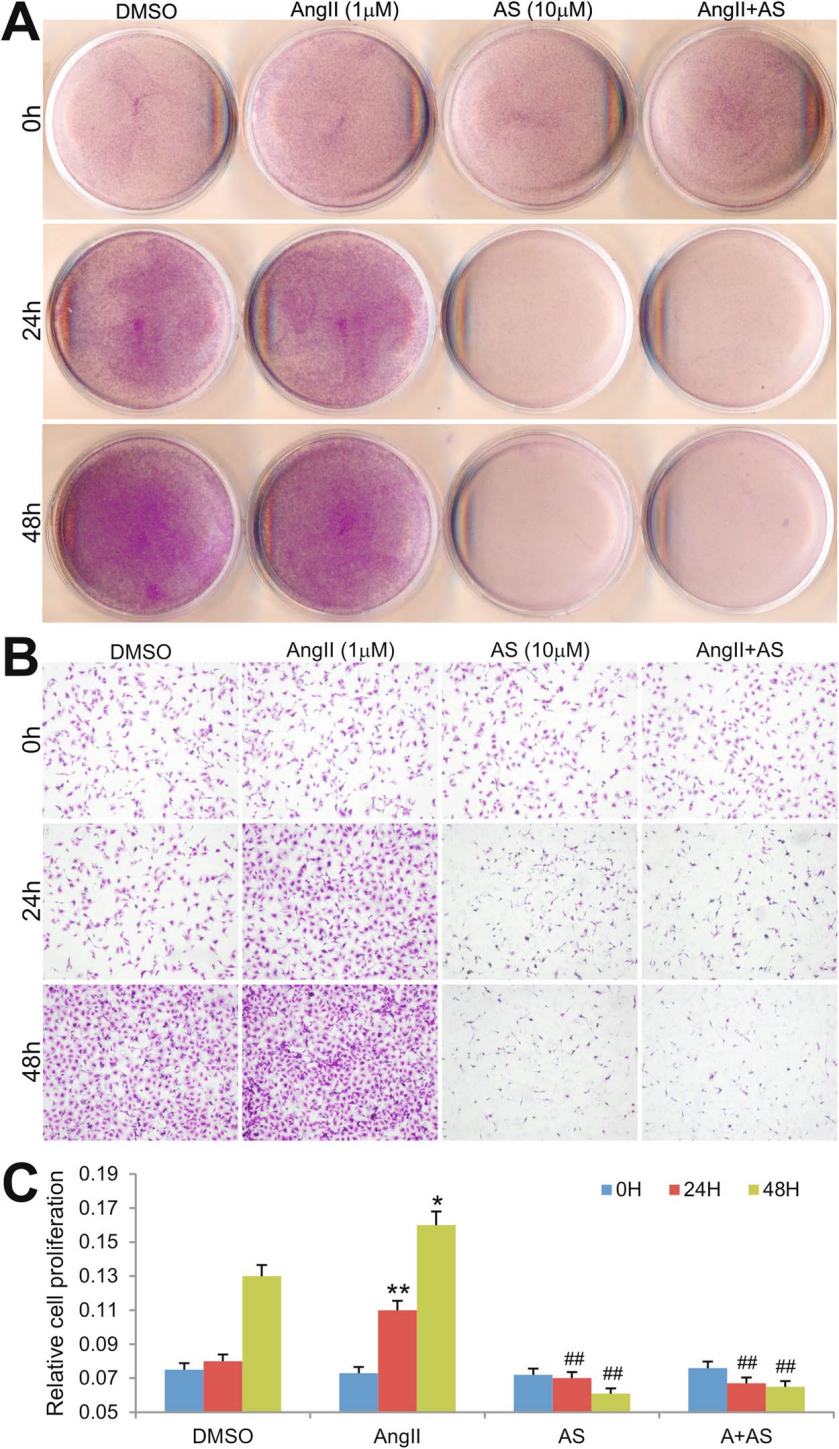
PI3Kγ inhibitor AS605240 suppresses NIH3T3 fibroblast cell proliferation. Subconflent NIH3T3 cells were treated with AS605240 (AS, 10 µM), angiotensin II (AngII, 1 µM), AngII (1 µM) and AS (10 µM) (AngII + AS), or DMSO control. Cell proliferation status was determined by Crystal violet staining, and documented by macrographic imaging (**A**) and under a bright field microscrope (**B**) at the indicated time points. The stained cells were dissolved for OD reading and quantitatively determined at A_590nm_ (**C**). The assays were performed in three independent batches of experiments. Representative results are shown. ^*^p < 0.05 and ^**^p < 0.01, compared with that of the control group; ^##^p < 0.01, compared with that of the Ang II treatment group.

We investigated the potential mechanism underlying the renal protective effect of PI3Kγ blockade in Ang II-induced renal damage. It was reported that PI3K promotes cell proliferation of murine hepatic stellate cells^47^. We sought to determine whether the inhibition of PI3Kγ signaling would suppress fibroblast cell proliferation. Using Crystal violet staining assay, we found that Ang II was able to stimulate NIH3T3 cell proliferation at 24 h and 48 h time points, which was effectively blocked by PI3Kγ inhibitor AS605240 (at 10 µM) (Fig. 5A,B). Quantitative analysis indicated that growth rate of cells treated with Ang II increased at 24 h and 48 h (p < 0.01 and p < 0.05, respectively), whereas the cells treated with AS605240 exhibited a significant reduction in cell survival rate compared to Ang II group (p < 0.01) (Fig. 5C).

**Figure 6:**
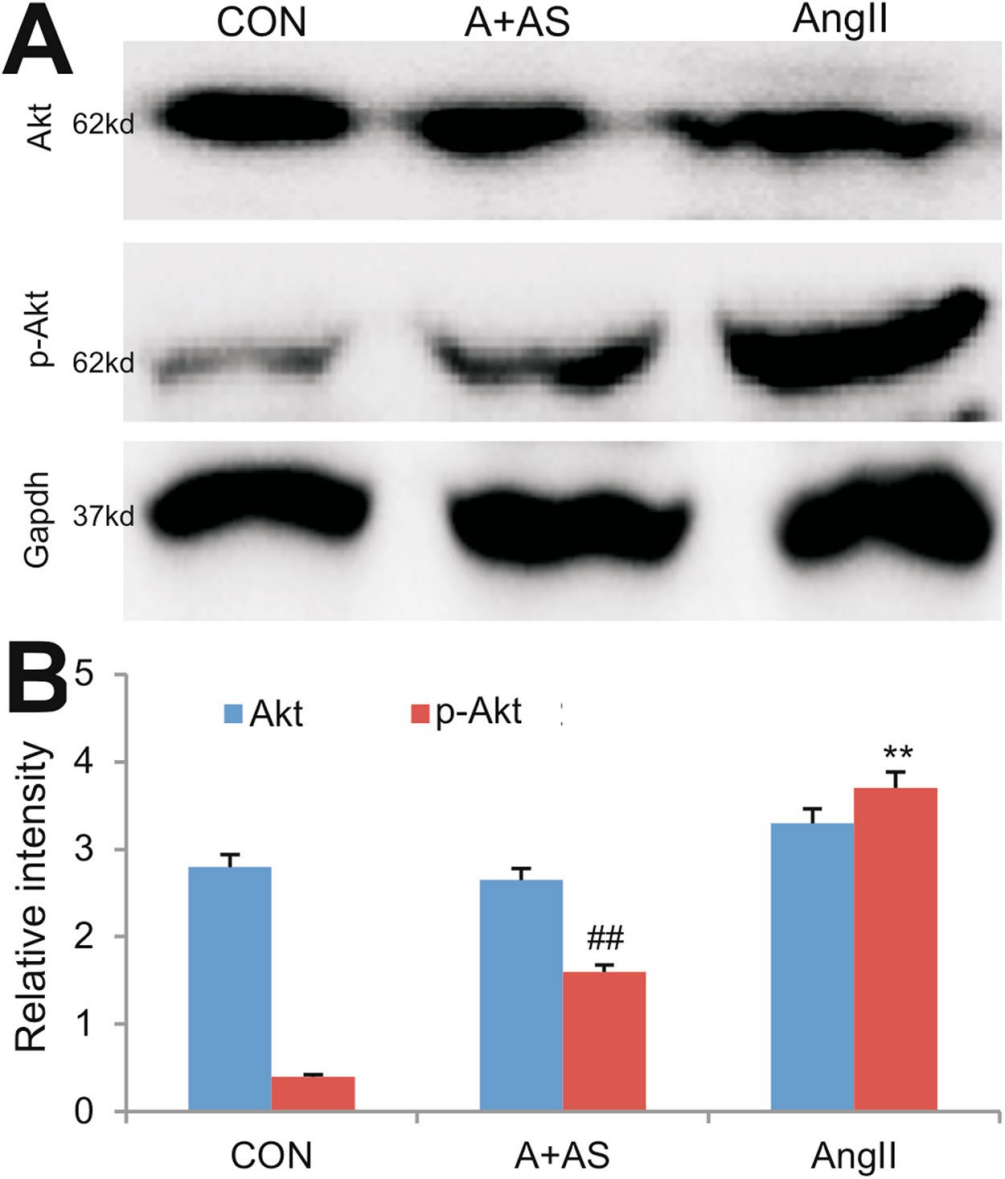
Inhibition of PI3Kγ blocks Ang II-induced activation of Akt signaling in NIH3T3 fibroblasts. Subconfluent NIH-3T3 cells were serum-starved for 24 hours, and then treated with Ang II, AS605240 + Ang II, or DMSO control in 10% FBS for 48 h. Total cell lysate was prepared and subjected to SDS-PAGE and Western blotting with Akt, p-Akt, or Gapdh antibody (**A**). Results are the representative for three independent experiments. The intensities of the blotting results were quantitatively determined by using Image Pro Plus 6.0 (**B**). ^**^p < 0.01, compared with that of the control group; ^##^p < 0.01, compared with that of the Ang II treatment group.

To examine a potential mechanism through which inhibition of PI3Kγ signaling may mediate antifibrogenic response, we also assessed the effect of blocking PI3Kγ on the Ang II-activated Akt signaling pathway. When subconfluent NIH3T3 cells were serum-starved for 24 h, and then treated with Ang II and/or AS605240, or DMSO control for 48 hrs. We found that, while the total Akt protein levels were similar among the three groups, the phosphorylated Akt (p-Akt) level was significantly increased in Ang II-stimulated cells, which was effectively blocked by PI3Kγ inhibitor AS605240 (Fig. 6A). Quantitative analysis confirmed that PI3Kγ inhibitor AS605240 was able to significantly inhibit Ang II-induced phosphorylation of Akt (p-Akt) (p < 0.01) (Fig. 6B). Collectively, these results suggest that PI3Kγ-induced activation of Akt may be at least in part responsible for cell survival and proliferation in fibroblasts.

### Inhibition of PI3Kγ signaling mitigates Ang II-induced expression of proinflammatory cytokines in kidney injury

**Figure 7:**
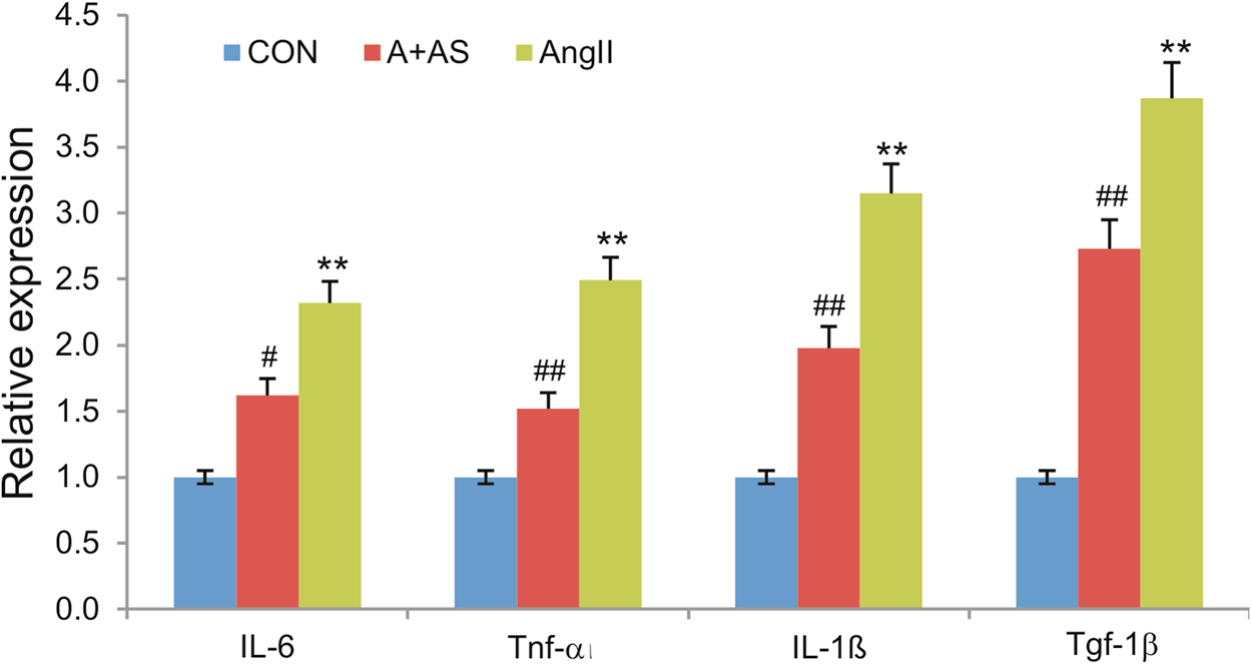
Inhibition of PI3Kγ signaling mitigates Ang II-induced expression of inflammatory cytokine genes in the kidney. Total RNA was isolated from the kidney tissues retrieved from Ang II-treated mice with or without AS605240 (n = 5 per group) and subjected to TqPCR analysis for the expression of mouse IL-6, Tnf-α, IL-1β and Tgf-1β. All reactions were done triplicate. All samples were normalized with Gapdh expression levels. ^**^p < 0.01, compared with that of the control group; ^#^p < 0.05 and ^##^p < 0.01, compared with that of the Ang II treatment group.

Lastly, we examined the effect of PI3Kγ blockade on the expression of pro-inflammatory cytokines that are involved in the pathogenesis of kidney injury. Total RNA was isolated from the retrieved mouse kidney tissues and subjected to qPCR analysis. We found that the expression of interleukin-6 (IL-6), tumor necrosis factor-α (Tnf-α), interleukin-1β (IL-1β), and transforming growth factor-β1 (Tgf-β1) was upregulated significantly in the mice treated with Ang II (p < 0.01) (Fig. 7). On the contrast, the Ang II-upregulated expression of IL-6, Tnf-α, IL-1β, and Tgf-β1 was significantly attenuated in the mice co-treated with Ang II and AS605240 (Fig. 7). Thus, these *in vivo* results further suggest that PI3Kγ signaling blockade may mitigate Ang II-induced expression of inflammatory cytokines during kidney injury.

## Discussion

CKD is a prevalent life-threatening disease frequently associated with hypertension, progressive tubulointerstitial fibrosis, and eventual renal failure^10,48–50^. Tubulointerstitial fibrosis is one of the most important factors for the development of chronic renal failure^20^. While the molecular mechanisms of underlying fibrosis are not fully understood, it is generally recognized that the activation of fibroblasts is a crucial stage of this process^20,51,52^. Here, through both *in vitro* and *in vivo* studies we demonstrate that PI3Kγ signaling may play an important role in mediating Ang II-induced the activation and cell proliferation of fibroblasts, up-regulation of myoblast formation, and the expression of fibrosis-related genes and inflammatory cytokines in mouse kidney tissues and/or NIH3T3 fibroblasts *in vivo* and/or *in vitro*, all of which may contribute to the development and progression of renal fibrogenesis. Conversely, a blockade of PI3Kγ signaling with AS605240 effectively mitigates Ang II-induced renal fibrogenic phenotypes *in vivo* and *in vitro*.

It has been well-recognized that abnormal activation of RAS plays a central role in the initiation and progression of CKD^22,52–54^. It was reported that Ang II can promote the phenotypic change of fibroblasts to myofibroblasts (α-Sma-positive cells)^10,48^; and these activated fibroblasts proliferated and invaded the periglomerular and peritubular spaces, contributing to matrix deposition in the tubulointerstitial area^55^. Cultured renal interstitial fibroblasts were shown to express AT1 receptors, and after Ang II stimulation there was an increase in cell proliferation, and expression and synthesis of ECM proteins such as fibronectin^55^. However, detailed mechanism of this process is currently unknown. Ang II acts through two types of receptors, angiotensin type 1(AT1) and angiotensin type 2 (AT2). It is generally accepted that AT1 regulates blood pressure, cell proliferation, and the production of cytokines and ECM proteins^55^. Accordingly, AT1 blocker (ARB) was shown to ameliorate the progression of chronic kidney disease^56^.

PI3Kγ belongs to the Class IB PI3Ks of the Class I PI3Ks and are activated by G-protein-coupled receptors^25^. Other studies and our results strongly suggest that inhibition of PI3K activity may effectively reduce fibrogenesis in many tissues including kidneys. Inhibition of fibrogenesis was associated with reduced α-SMA expression and collagen production^47^. Since activated fibroblasts (i.e., α-SMA-positive cells) are the principal effector cells responsible for extracellular matrix production in the fibrotic kidney, their activation is considered as a key event in the pathogenesis of tubulointerstitial fibrosis^18,19^. One possible mechanism involved in fibrogenesis may be that PI3K-induced Akt activation may lead to cell survival and proliferation. Sustained activation of Akt was shown to induce significant cell proliferation^47^. In this study, we demonstrated that inhibition of PI3Kγ activity in NIH3T3 cells led to a substantial decrease of Ang II-induced Akt activation *in vitro*. We also demonstrated that inhibition of PI3Kγ could reduce Ang II-induced cell proliferation. Therefore, an inhibition of PI3Kγ and its downstream mediators would significantly impact cell growth and proliferation^47^.

Inhibition of PI3Kγ signaling may also suppress the activation of fibroblasts and subsequent production of profibrogenic factors^16,20^. In our study, we found that expression of α-Sma was decreased when PI3Kγ signaling was inhibited both *in vitro* and *in vivo*. Moreover, the inhibition of PI3Kγ led to reduction in collagen expression and deposition, and production of profibrogenic growth factors, such as fibronectin and connective tissue growth factor both *in vitro* and *in vivo*^57,58^. Therefore, it is conceivable that inhibition of PI3Kγ signaling may impact its fibrogenic potential at multiple cellular levels, from cell activation and proliferation to promotion of profibrogenic factors production^47,57,58^. In this work, we only examine the effect of the PI3Kγ inhibitor AS605240. Given the rapid development of numerous PI3K small molecule inhibitors, it is conceivable that other inhibitors may be more effective and/or specific than AS605240. Thus, one of the future directions is to characterize more PI3K inhibitors for their effect on alleviating tubulointerstitial fibrosis and/or CDK.

Lastly, our results indicate that blockade of PI3Kγ signaling effectively diminishes Ang II-induced expression of proinflammatory cytokines, such as of IL-6, Tnf-α, IL-1β, and Tgf-β1, in the kidney. It was reported that Ang II was able to induce the expression of proinflammatory cytokines^59,60^. This is mechanistically relevant because proinflammatory cytokines Tnf-α, IL-6, and IL-1β were implicated in the pathogenesis of Ang II-induced organ damage^61^, and Tgf-β1 was shown to function as a downstream mediator of Ang II-induced tubulointerstitial fibrosis^62–64^.

In summary, we demonstrate that PI3Kγ inhibitor AS605240 effectively mitigates Ang II-induced increases in serum creatinine and blood urea nitrogen, renal interstitial collagen deposition, the accumulation of ECM proteins and the expression of α-SMA and fibrosis-related genes *in vivo*. Mechanistically, fibroblast cells treated with AS605240 exhibits a significant reduction in cell proliferation and phosphorylated Akt upon Ang II stimulation. Furthermore, we demonstrate that the Ang II-upregulated expression of IL-6, Tnf-α, IL-1β and Tgf-β1 is significantly attenuated in the mice treated with PI3Kγ inhibitor AS605240. Therefore, targeted PI3Kγ inhibition may be explored as a novel therapeutic approach to the clinical management of CKD.

## Electronic supplementary material

Supplementary Table 1
