# Supplementary material for: A blockade of PI3Kγ signaling effectively mitigates angiotensin II-induced renal injury and fibrosis in a mouse model
Source: Sci Rep. 2018 Jul 20;8:10988. doi: 10.1038/s41598-018-29417-3 (PMC6054654; doi:10.1038/s41598-018-29417-3)
Supplement: Supplementary file 1 — Supplementary Table 1 [file 41598_2018_29417_MOESM1_ESM.docx]

**A blockade of PI3Kγ signaling effectively mitigates angiotensin II-induced renal injury and fibrosis in a mouse model**

**Running Title**: **Protective role of PI3K inhibition in chronic kidney disease**

Xinyi Yu^1,2^, Yunfeng Xia^1^, Liyi Zeng^2,3^, Xi Zhang^1^, Liqun Chen^1,2^, Shujuan Yan^2,4^, Ruyi Zhang^2,4^, Chen Zhao^1,2^, Zongyue Zeng^2,4^, Yi Shu^2,4^, Shifeng Huang^1,2^, Jiayan Lei^1,2^, Chengfu Yuan^2,5^, Linghuan Zhang^2,4^, Yixiao Feng^1,2^, Wei Liu^1,2^, Bo Huang^2,3,6^, Bo Zhang^2,7^, Wenping Luo^2,8^, Xi Wang^2,4^, Hongmei Zhang^2,8^, Rex C. Haydon^2^, Hue H. Luu^2^, Tong-Chuan He^2^*, and Hua Gan^1^*

^1^ Departments of Nephrology, Orthopaedic Surgery, Cardiology, General Surgery, and Clinical Laboratory Medicine, the First Affiliated Hospital of Chongqing Medical University, Chongqing 400016, China

^2^ Molecular Oncology Laboratory, Department of Orthopaedic Surgery and Rehabilitation Medicine, The University of Chicago Medical Center, Chicago, IL 60637, USA

^3^ Department of Infection Control, Zhuzhou Central Hospital, and the Affiliated Zhuzhou Hospital of Xiangya Medical College of Central South University, Zhuzhou, China

^4^ Ministry of Education Key Laboratory of Diagnostic Medicine and School of Laboratory Medicine, and the Affiliated Hospitals of Chongqing Medical University, Chongqing 400016, China

^5^ Department of Biochemistry and Molecular Biology, China Three Gorges University School of Medicine, Yichang 443002, China

^6^ Department of Clinical Laboratory Medicine, the Second Affiliated Hospital of Nanchang University, Nanchang 330031, China

^7^ Key Laboratory of Orthopaedic Surgery of Gansu Province and the Department of Orthopaedic Surgery, the Second Hospital of Lanzhou University, Lanzhou, 730030, China

^8^ Chongqing Key Laboratory for Oral Diseases and Biomedical Sciences, and the Affiliated Hospital of Stomatology of Chongqing Medical University, Chongqing 401147, China

* Corresponding authors

**CORRESPONDENCES**

T.-C. He, MD, PhD

Molecular Oncology Laboratory

The University of Chicago Medical Center

5841 South Maryland Avenue, MC 3079

Chicago, IL 60637, USA

Tel. (773) 702-7169

Fax (773) 834-4598

E-mail: [tche@uchicago.edu](mailto:tche@uchicago.edu)

Hua Gan, MD

Department of Nephrology

The First Affiliated Hospital of Chongqing Medical University

Chongqing 400016, China

Tel. (86) 23-8901-2019

E-mail: [ghzxgckd@163.com](mailto:ghzxgckd@163.com)

**Supplementary Table 1. qPCR Primers**

| **Gene (mouse)** | **Sequence** | |
| --- | --- | --- |
| IL-6 | Forward | 5’-GAGGATACCACTCCCAACAGACC-3’ |
|  | Reverse | 5’-AAGTGCATCATCGTTGTTCATACA-3’ |
| Tnf-α | Forward | 5’-CATGAGCACAGAAAGCATGATCCG-3’ |
|  | Reverse | 5’-AAGCAGGAATGAGAAGAGGCTGAG-3’ |
| Tgf-1β | Forward | 5’-CAACAATTCCTGGCGTTACCTTGG-3’ |
|  | Reverse | 5’-GAAAGCCCTGTATTCCGTCTCCTT-3’ |
| IL-1β | Forward | 5’-CTTCAGGCAGGCAGTATCACTCAT-3’ |
|  | Reverse | 5’-TCTAATGGGAACGTCACACACCAG-3’ |
| Fibronectin | Forward | 5’-AATGGAAAAGGGGAATGGAC-3’ |
|  | Reverse | 5’-CTCGGTTGTCCTTCTTGCTC-3’ |
| Ctgf | Forward | 5’-GTGCCAGAACGCACACTG-3’ |
|  | Reverse | 5'-CCCCGGTTACACTCCAAA-3’ |
| Col1a1 | Forward | 5’-GCTCCTCTTAGGGGCCACT-3’ |
|  | Reverse | 5’-CCACGTCTCACCATTGGGG-3’ |
| α-Sma | Forward | 5’-TGAGGAGACTTGCCTGGTG-3’ |
|  | Reverse | 5’- GCATTTGTGGTTGGGTCAG-3’ |
| Gapdh | Forward | 5’-GCCTCGTCCCGTAGACAAAA-3’ |
|  | Reverse | 5’-TTCCCATTCTCGGCCTTGAC-3' |
